# Carbon quantum dot-based fluorometric detection of nitrosamine impurities in active pharmaceutical ingredients

**DOI:** 10.1039/d5na00490j

**Published:** 2025-08-19

**Authors:** Gayathri Loganathan, Pirangi Srikanth, Khaja Moinuddin Shaik, Sukhendu Nandi

**Affiliations:** a Department of Pharmaceutical Analysis, National Institute of Pharmaceutical Education and Research S.A.S. Nagar Mohali 160062 Punjab India sukhendu@niper.ac.in

## Abstract

Nitrosamines are genotoxic, mutagenic impurities and are widely encountered in the global landscape of the pharmaceutical industry. There is a need for rapid detection of nitrosamines in a pharmaceutical product. Here, we report the synthesis of carbon quantum dots (CQDs) using a readily available carbon precursor. These CQDs showed attractive fluorescence properties and were employed to fabricate a portable device for the fluorometric detection of nitrosamine impurities in a drug matrix.

## Abstract 2

A biosensor that uses carbon quantum dots enables precise detection and measurement of nitrosamine impurities, representing a significant advancement in analytical technology for quality control of active pharmaceutical ingredients (APIs).

## Graphical abstract

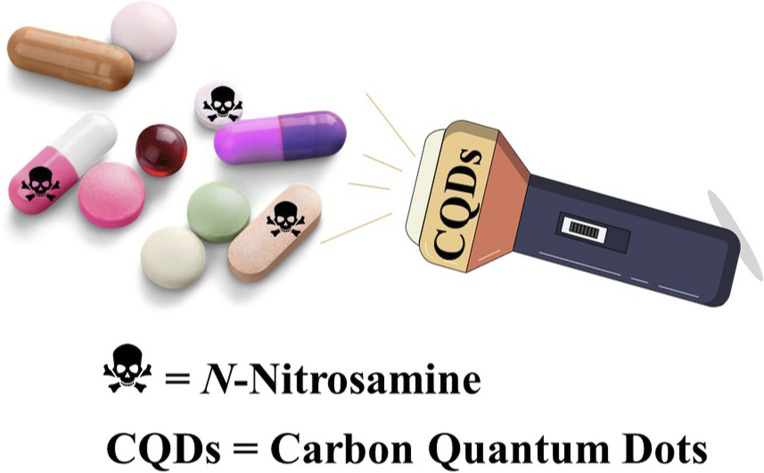

Nitrosamines or *N*-nitrosoamine compounds are a class of compounds with nitroso groups bonded to amines.^1^ They are a major concern in the global regulatory landscape of the pharmaceutical industry since they are potent carcinogens and mutagens. Nitrosamines are widespread in our environment, such as in our drinking water; tobacco; beverages; and foods, including cured and grilled meats; dairy products; and drugs.^2–5^ Recently, nitrosamines have attracted increased attention due to their detection in unacceptable levels in various drug products.^6^ The International Agency for Research on Cancer (IARC), an intergovernmental agency of the World Health Organization (WHO) of the United Nations (UN), has classified nitrosamines as “2A-probably carcinogenic to humans,” and according to the M7(R1) guideline of the International Council for Harmonisation of Technical Requirements for Pharmaceuticals for Human Use (ICH), nitrosamine impurities are classified as a “cohort of concern,” which means a group of high potency mutagenic carcinogens. Otto N. Witt, a German scientist, initially synthesized nitrosamine in 1874 by reacting secondary or tertiary amines with HNO_2_ and its ethers.^1,7,8^ However, in July 2018, major pharmaceutical companies recalled their valsartan drug products from the market due to an unacceptable level of a carcinogenic nitrosamine impurity called *N*-nitrosodimethylamine (NDMA).^9–15^ Additionally, combination medications that contained valsartan, irbesartan, and losartan with amlodipine and hydrochlorothiazide (HCTZ) were withdrawn from market distribution because of a United States Food and Drug Administration (US FDA) order.^16^ Thereafter, these impurities were found in other non-sartans such as pioglitazone (Jan. 2019), ranitidine (Sept. 2019),^17^ metformin (Nov. 2019),^17^ rifampicin (Aug. 2020), nizatidine (April 2020), and varenicline (Sept. 2021)18. The US FDA database shows that, because of nitrosamine impurities beyond the acceptable intake levels (26.5 ng per day),^19^ companies have recalled more than 1400 products from the market.^17^ In September 2020, the US FDA released guidelines for the control of nitrosamines in drug products. These guidelines, which were recently updated in September 2024, requested marketing authorization holders to review their manufacturing process, active pharmaceutical ingredient (API), and finished products to identify the presence of nitrosamine impurities.^7,20^ Regulatory agencies such as the US FDA, European Medicines Agency (EMA), ICH, and WHO constantly monitor the possibility of nitrosamine impurities in a drug product by providing guidance and regulatory updates to all drug manufacturers. Mitigating the probability of nitrosamine impurities in food and pharmaceutical products requires a robust, rapid, and reliable analytical method with high accuracy and precision. Some existing methods for detecting NDMA and NDEA are based on LC-MS/MS^21,22^ and GC-MS/MS.^23–26^ Although these methods are highly sensitive, with a detection limit at the ppb level, they are still inefficient as they are highly time-consuming; require skilled personnel and specialized equipment; and often involve complicated pre-treatment, including filtration and extraction.^27,28^ Therefore, there is a need for an alternative non-chromatographic toolkit for rapid and highly accurate detection of nitrosamine impurities.^29^

We designed a CQD-based fluorescence biosensor to detect and quantify nitrosamine impurities. CQDs, a rising star in the carbon nanostructure family, have attracted enormous attention because of their exceptional luminescence property,^30,31^ high-quantum yield,^32–34^ and broad-spectrum luminescence,^35^ making them useful for several sensing^36,37^ and imaging applications.^35,38,39^ Another advantage of CQDs is that they can be easily synthesized from readily available carbon precursors,^40,41^ and their photophysical properties can be tailored accordingly depending on the precursor.^42^ Herein, we report the design and synthesis of CQDs that showed very attractive fluorescence properties and were employed for detecting NDMA, a representative example of the nitrosamine class of impurities.

## Results and discussion

### Syntheses and characterizations of CQDs

**Fig. 1:**
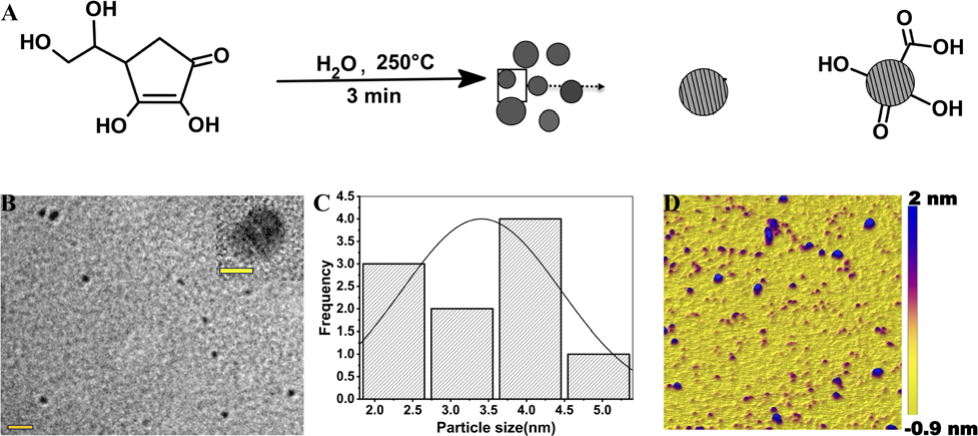
Synthesis and characterization of CQDs. (A) Synthetic scheme. (B) High-resolution transmission electron microscopy (HR-TEM) image of CQDs (scale bar is 20 nm); inset is the HR-TEM image of a single CQD (scale bar is 5 nm) showing the crystal planes. (C) Particle size distribution histogram of the as-synthesized CQDs obtained from HR-TEM. (D) AFM image of CQDs on mica.

**Fig. 2:**
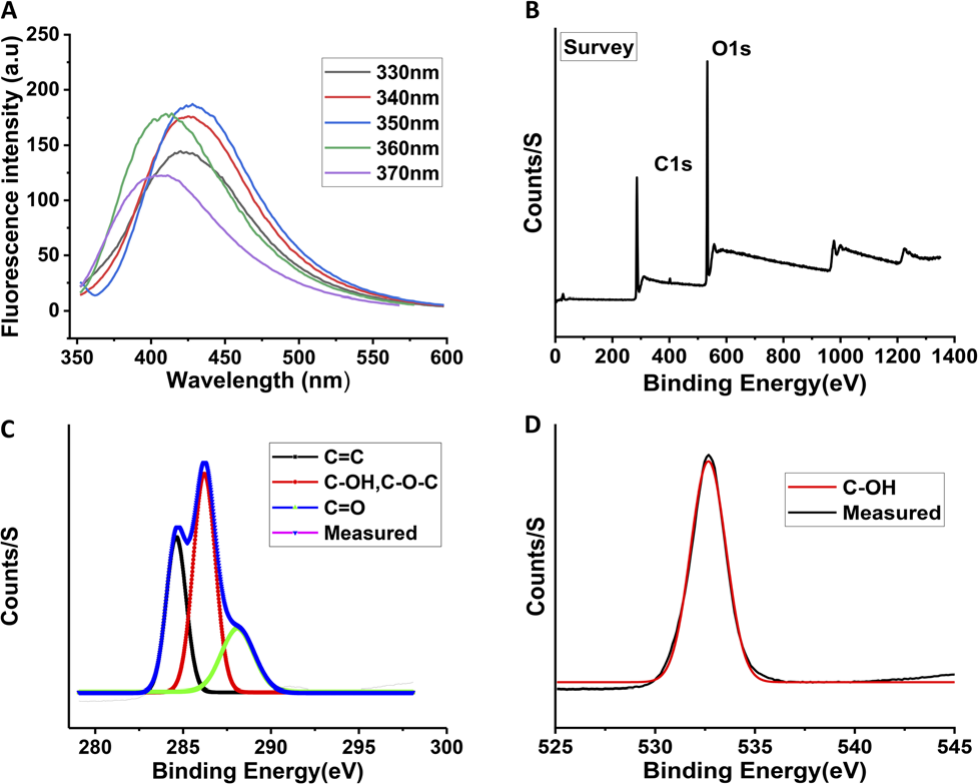
(A) Excitation wavelength-dependent luminescence of the as-synthesized CQDs in methanol solution; XPS spectra of the prepared CQDs. (B) Survey-scan spectrum, (C) C 1s, and (D) O 1s.

Fig. 1A shows the schematic representation of the synthesis of carbon quantum dots starting from ascorbic acid as a precursor using a hydrothermal reaction. The reaction was performed in water at 250 °C in a Teflon-capped sealed tube, eventually forming CQDs. The as-synthesized CQDs have a graphite core structure, which was confirmed by HR-TEM. The HR-TEM image shows lattice fringes with an interplanar spacing of 0.21 nm that corresponds to the [110] facet of the graphitic core structure of the as-synthesized CQDs (Fig. 1B). The size distribution of the as-synthesized CQDs was measured using HR-TEM (Fig. 1B), showing a particle size distribution from 1.8 nm to 5.2 nm with an average size distribution of around 3.5 nm (Fig. 1C). The AFM image of the as-synthesised CQDs and the corresponding topographical height distribution revealed an average particle thickness of 3.9 nm (Fig. 1D). The distribution of height obtained from the AFM image is depicted in Fig. S1. Fig. 2A represents the luminescence map of the CQDs. Notably, the as-synthesized CQDs showed an excitation wavelength-dependent luminescence property, with an emission maximum at 427 nm when excited at 350 nm. The obtained quantum yield of the CQDs in water, using quinine sulphate as reference, was 3%. The fluorescence stability of the CQDs as a function of temperature was measured (Fig. S2A–C). With an increase in temperature, a decrease in the fluorescence intensity of CQDs was observed, as depicted in Fig. S2A. However, after heating, if the solution of the CQDs was cooled down to room temperature, their fluorescence intensity at the excitation maximum was restored, as depicted in Fig. S2C. A similar phenomenon has also been observed by other groups.^43–45^ The as-synthesized CQDs contain dense population of several oxygen-containing functional groups on their surfaces. XPS confirmed the existence of different oxygen-containing functional groups at the surface of the CQDs. XPS survey signals of C 1s and O 1s appeared at 285.0 and 530.6 eV, respectively (Fig. 2B). The XPS survey scan of the CQDs shows that the CQDs mainly comprised carbon and oxygen, *i.e.*, C 1s (37.97%) and O 1s (62.03%), respectively. The absence of any other element in the overall XPS survey scan further reflects the purity of the CQDs. A well-fitted C 1s high-resolution spectrum (Fig. 2C) shows the signals of the C=C bond at 284.6 eV, C–OH/C–O–C bond at 286.2 eV, and C=O bond at 288 eV. The O 1s spectrum (Fig. 2D) shows the C–OH, C–O–C, and C=O signals at 532.6 eV. FT-IR spectroscopy further confirmed the existence of dense oxygen-containing functional groups on the surface of the CQDs (see Fig. S3). The appearance of vibrational bands at 3524, 3406, and 3311 cm^−1^ is firmly attributed to C–OH stretching, 1675 cm^−1^ to C=C stretching, 1111 cm^−1^ to C=O stretching, 1313 cm^−1^ to C–H bending, 1753 cm^−1^ to C–O–C stretching, and 1022 cm^−1^ to C–OH bending.

### Quenching of fluorescence from CQDs with NDMA

**Fig. 3:**
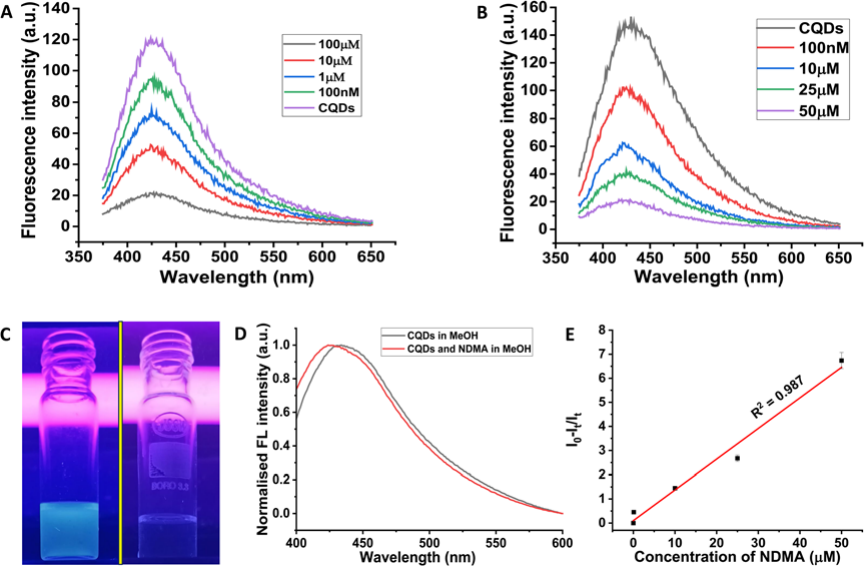
(A) Fluorescence spectra of carbon quantum dots (CQDs) upon the addition of different concentrations of TEMPO radicals. (B) Decrease in fluorescence of the CQDs mediated by NO radicals after the addition of various concentrations of NDMA. (C) A digital photograph shows the quenching of CQDs fluorescence after adding NDMA under a UV lamp. (D) The shift in fluorescence spectra from CQDs before and after the addition of NDMA. (E) A graph illustrating the fluorescence quenching efficiency, calculated as (*I*_0_ − *I*_t_)/*I*_t_ plotted against the concentration of NDMA.

To investigate whether the fluorescence of the CQDs is sensitive to the concentration of free radicals, we obtained a titration curve in which the concentration of the CQDs in methanol was kept constant in every case with a gradual increase in TEMPO (2,2,6,6-tetramethyl-1-piperidinyloxy) radical concentration, as shown in Fig. 3A. TEMPO is a stabilized NO radical widely used in electron paramagnetic resonance spectroscopy.^46^ Interestingly, we observed that as the concentration of TEMPO radicals increased, the fluorescence of the synthesized carbon quantum dots (CQDs) was quenched when excited at 350 nm. This suggests that the fluorescence of the synthesized CQDs may be sensitive to the presence of free radicals in the solution. This also indicates that CQDs, such as those synthesized, can detect nitrosamine impurities after the photochemical degradation of nitrosamine impurities to NO radicals. Fig. 3B represents the quenching of fluorescence of the CQDs when they were co-incubated with NDMA, a representative example of nitrosamine impurities. Then, the mixture was subjected to UV treatment for 2 h. The photochemistry of nitrosamines is well established, and it is known that they produce NO radicals after photolytic degradation,^18^ leading to the quenching of CQD fluorescence when such *in situ* generated NO reacts with CQDs. Fig. 3C represents the corresponding digital fluorescence image for CQDs under a UV lamp before (left) and after (right) the addition of NDMA, clearly representing the dramatic visual demonstration of a significant decrease in the fluorescence from CQDs. From the digital image, NO radical-induced reduction in the fluorescence of CQDs was very apparent. Fig. 3D represents the shifting of fluorescence from the CQDs in methanol before and after incubation with NDMA under a UV lamp when the CQDs were excited at 350 nm in both cases. Such a shift in the emission maximum clearly indicates that there is an interaction of UV-mediated *in situ* generated NO radicals with CQDs. However, the detailed mechanism of such interaction needs to be studied further. The degree of fluorescence quenching with NO radical concentration mediated by NDMA is depicted in Fig. 3E, underscoring the relation between the quenching of the fluorescence of the CQDs and NDMA concentrations. This indicated an excellent degree of a linear relationship (*R*^2^ = 0.987) between CQD fluorescence and NDMA concentration, with a detection threshold of 10 μM.

### Fabrication of a CQD-based portable biosensor

**Fig. 4:**
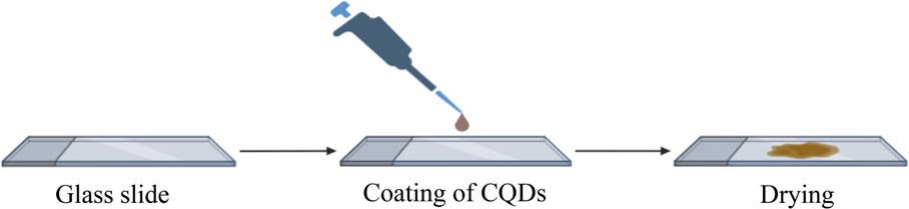
Schematic representation of the fabrication of a portable biosensor based on CQDs.

We fabricated a biosensor device to translate this concept into a feasible, portable, fluorescent-based toolkit for detecting and quantifying nitrosamine impurities, as shown in Fig. 4. For biosensor fabrication, we drop-cast a methanol solution of the CQDs onto a cleaned glass slide and dried it under vacuum, forming a thin layer of the CQDs adhered onto the glass surface.

**Fig. 5:**
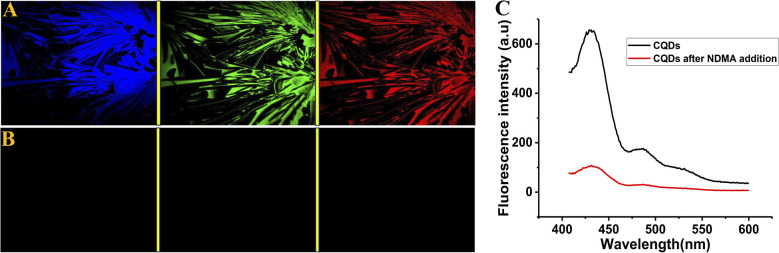
Fluorescence microscopy images of the thin film of the CQDs before (A) and after (B) NDMA addition, by subjecting the film to excitation at 405 nm with emission filter EM 450 (blue), excitation at 488 nm with emission filter EM 505 (green), and excitation at 543 nm with emission filter EM 595 (red). (C) Quenching of the fluorescence of a glass-supported solid film of the CQDs before and after the addition of NDMA.

Fig. 5 represents the corresponding confocal fluorescence microscopy image of the thin film of the CQDs onto a solid glass surface in three different distinct excitation-emission wavelengths before (Fig. 5A) and after the addition of NDMA, followed by subjecting the thin film to UV light (Fig. 5B), leading to the quenching of CQD fluorescence. Fig. 5 depicts the real-time observation of a decrease in fluorescence with the addition of NDMA, demonstrating that such a portable device can detect and quantify nitrosamine impurities in a photo-stable active pharmaceutical ingredient (API) or excipient. To further confirm such a quenching phenomenon, we monitored the fluorescence from the solid biosensor devices before and after the addition of NDMA, followed by UV treatment, using a spectrofluorometer. A decrease in NO-mediated fluorescence intensity further confirmed that such a portable toolkit could detect *N*-nitrosodimethylamine in any pharmaceutical ingredients that do not undergo photolytic degradation to yield radicals.

### Validation of the method

**Fig. 6:**
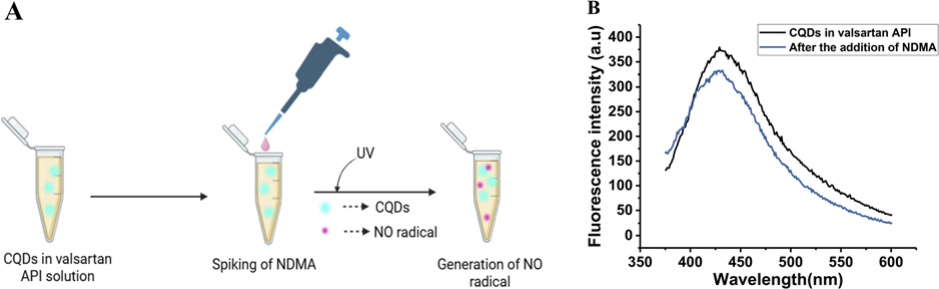
(A) Schematic representation of NDMA spiking into a methanol solution of losartan. (B) Quenching of fluorescence from CQDs in a losartan drug matrix before and after the addition of NDMA.

Method validation is the process of showing that the developed method is suitable for its intended use.^47^ We further applied this method to detect nitrosamines in an API by spiking NDMA in that particular API. Here, the validation was carried out by spiking 100 μM NDMA in a 1000 ppm valsartan API, as shown in Fig. 6A. Fluorescence was monitored immediately after the coincubation of the valsartan API with the CQDs and 5 hours after UV treatment. The corresponding fluorescence spectrum before and after UV treatment is depicted in Fig. 6B. Fig. 6B shows the quenching of the CQDs' fluorescence before and after the addition of NDMA when excited at 350 nm.

## Conclusion

In summary, using a hydrothermal reaction, we successfully synthesized CQDs through a top-down approach with ascorbic acid as a precursor. The fluorescence of the as-synthesized CQDs is sensitive to free radicals and was employed to detect and quantify nitrosamine impurities using NDMA as an example. A solid-based biosensor was fabricated using a drop-casting technique to develop a portable fluorescence-based toolkit device to detect and quantify nitrosamine impurities. The feasibility of the biosensor for detecting nitrosamine impurities was validated using valsartan as an API. Hence, the biosensor can be used as a portable device for quality control in small and medium pharmaceutical industries to eliminate the possibility of nitrosamine impurities in a pharmaceutical product.

## Conflicts of interest

The authors affirm that they have no identifiable personal or competing conflicts of interest that could have influenced the research presented in this article.

## Supplementary Material

NA-007-D5NA00490J-s001

## Data Availability

The data supporting this article have been included as part of the SI. See DOI: https://doi.org/10.1039/d5na00490j.
