# Supplementary material for: Carbon quantum dot-based fluorometric detection of nitrosamine impurities in active pharmaceutical ingredients
Source: Nanoscale Adv. 2025 Aug 19;7(20):6412–6. doi: 10.1039/d5na00490j (PMC12411933; doi:10.1039/d5na00490j)
Supplement: NA-007-D5NA00490J-s001 [file NA-007-D5NA00490J-s001.pdf]

Supplementary Information (SI) for Nanoscale Advances.
This journal is © The Royal Society of Chemistry 2025

Carbon quantum dot-based fluorometric detection of nitrosamine impurities in active pharmaceutical ingredients

Gayathri Loganathan, Pirangi Srikanth, Khaja Moinuddin Shaik and Sukhendu Nandi*

MATERIALS AND EQUIPMENT

L-Ascorbic acid (99.7%) extra pure was purchased from SRL chemicals, N-nitrosodimethylamine (NDMA) was purchased from Sigma Aldrich, 2,2,6,6-tetramethyl-1-piperidinyloxy radical (TEMPO) from Spectrochem.

SYNTHESIS OF CQDs

CQDs were prepared by one-pot synthesis using a hydrothermal method. 50mg of *L*-Ascorbic acid was taken in a 5 mL glass vial, and 100 µL of distilled water was added. The vial was closed tightly with Teflon tape. The glass vial was heated on a hot plate at 250° C for 2-3 minutes until it turned to a brownish-black mass. 300 µL of ethanol was added to the crude, and the mixture was sonicated for about 5 minutes until the carbon mass was disintegrated. The mixture was vortexed for 5 minutes and centrifuged at 10000 rpm for 10 minutes. The supernatant was taken and filtered further through 0.22 µM pore-sized filter paper. The filtrate was then dried under reduced pressure to get pure CQDS, which was then stored in an ampule vial.

FLUORESCENCE SPECTROSCOPY

The fluorescence spectra of the as-synthesized CQDs solutions were recorded on a CARY-Eclipse/ LS-50B spectrofluorometer. Quenching efficiency (QE) was calculated based on the Stern-Volmer equation as follows.

$$\mathbf{QE=(}\boldsymbol{I_0-I_t}\mathbf{)/}\boldsymbol{I_t}$$

Where I_0 corresponds to the initial fluorescence, and I_t corresponds to the fluorescence recorded after NDMA addition.

The fluorescence of carbon-coated glass slides was recorded using the front-face fluorescent spectroscopic technique. The sample was kept at an angle of 60° from the incident light. The measurement was taken by a CARY-Eclipse/ LS-50B spectrofluorometer using 370 nm as an excitation wavelength, and corresponding emission spectra were recorded from 400 to 600 nm.

High-resolution transmission electron microscopy (HR-TEM)

HR-TEM images for size distribution were recorded on an FEI Technai G2 F20 X29000. HR-TEM experiment for lattice fringes was recorded on a 200 kV JEOL JEM-2100F. For the HR-TEM measurement, 0.5 mg of as-synthesized carbon dots were dissolved in 500 µL of methanol, and 10 µL of the solution was placed upon an ultrathin carbon film-coated copper grid, dried at room temperature for 2 h, and imaged.

Atomic Force Microscopy (AFM)

was carried out with a Dimension 3100 SPM Digital Instruments (Veeco, NY, USA). The surface morphology of the CQDs was analysed by drop casting a methanol solution of CQDs having a concentration of 0.01 mg/mL, followed by drying in air. The thickness distribution of the nanoparticles was calculated by using NanoScope Analysis v 1.40 software, and the statistical distribution was plotted with OriginPro software (Version 8.5)

CONFOCAL LASER SCANNING MICROSCOPY (CLSM)

Confocal microscopy images were obtained with OLYMPUS FLUOVIEW FV1000 (Tokyo, Japan) equipped with argon and He–Ne lasers, and the images were taken with a 60×/1.4 oil immersion objective.

FOURIER TRANSFORM INFRARED SPECTROSCOPY (FTIR)

IR spectra were obtained using an FTIR (Perkin Elmer) instrument using an ATR-FTIR-based technique. The sample was run between 4000-400 cm^{-1}.

X-RAY PHOTOELECTRON SPECTROSCOPY (XPS)

XPS was conducted using ESCA LAB 250 XI, utilizing a Mg Kα non-monochromatic X-ray beam with a photon energy of 1253.6 Ev.

PREPARATION OF SAMPLE FOR VALIDATION OF NDMA

1mg of valsartan was taken and dissolved in 1 ml of methanol. To the prepared methanol solution of losartan, 100 μM NDMA was spiked and incubated under a UV lamp at 254nm

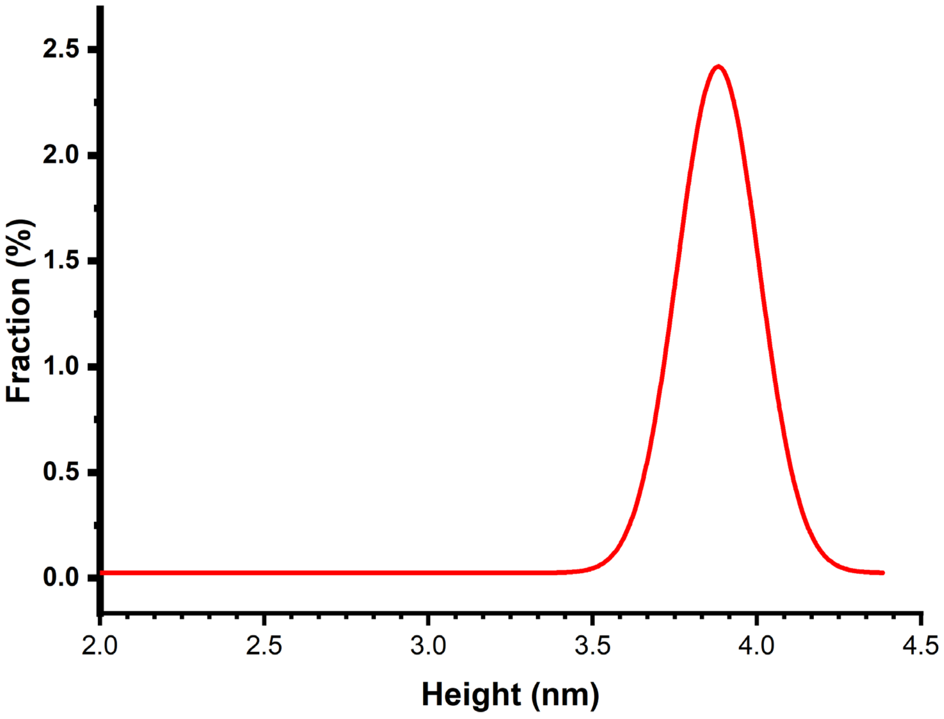

Figure S1: Height distribution of CQDs recorded by the AFM experiment.

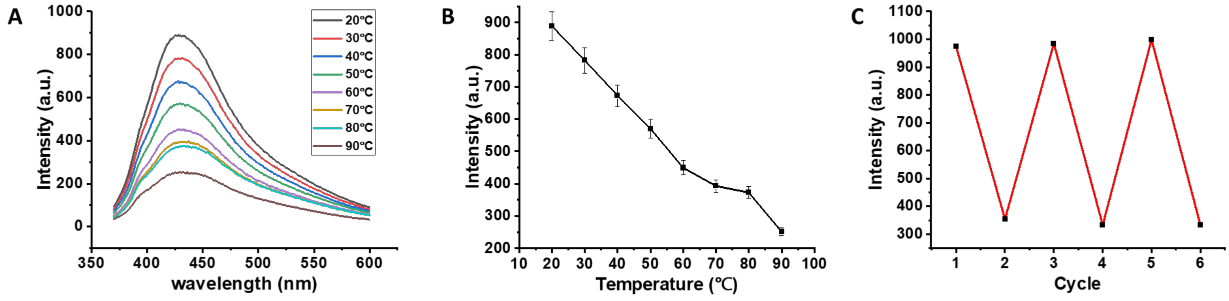

Figure S2: A: Quenching of FL intensity of CQDs in water with increasing temperature from 20 ^{0}C to 90 ^{0}C. B: Plot of FL intensity of CQDs in water at 350 nm excitation with Temperature. C: Cycling of fluorescence maxima of CQDS solution in water between T = 20 °C and T = 90 °C.

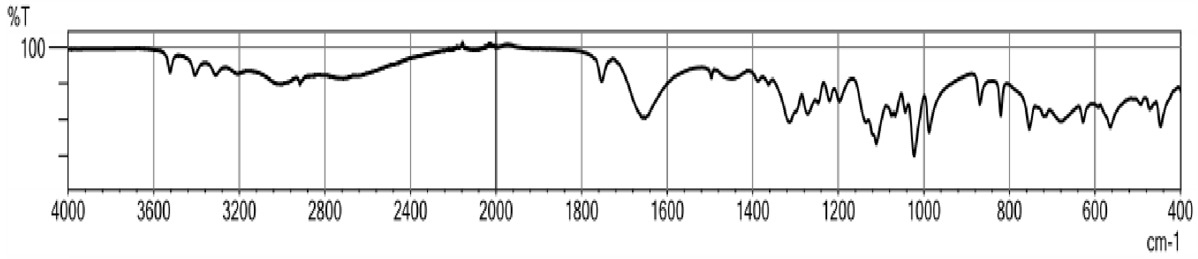

Figure S3: FT-IR spectrum of CQDs.
